# To the Editors of the Medical and Physical Journal

**Published:** 1808-07

**Authors:** E. Kentish

**Affiliations:** Bristol


### *To the Editors of the Medical and Physical Journal.*

GENTLEMEN,

IN the Journal for June 1808, p. 519, Dr. Wood, in his Reply to my Answer, changes the ground on which I formed my justification; he has now said, his paper was read in September 1792; consequently, it is within the scale of possibility, I might have either heard or seen it. This is evidently what Dr. Wood insinuates. In answer to which, I reply, that I was not a member of the Newcastle Medical Society at that time, consequently I did not hear it read. His paper was written on Typhus Fever, and I shall now assign the reason why I never read it. The remedy he most recommended was the nitrate of potass. In a conversation with the late Dr. Andrew Young, of Newcastle, he ridiculed in such a vein of sarcastic humour, the idea of curing fever with so much debility with this weapon, that I confess myself to have become prejudiced; and when he published his book, my prejudice was so strong that I never read it; at that time I was too much biased in favour of bark, wine, opium, &c. in fever, to give the impartial attention to Dr. Wood's dissertation which every work merits. This confession will explain to my readers, and apologize to Dr. Wood, for my not seeing his work. Laying aside this insinuation of the Doctor's, his answer does not bring forward any proof that his principles had been applied in the treatment of burns. It will appear extraordinary, that Dr. Wood's principles should have produced such an effect upon me, who neither heard nor saw them, and so little upon the secretary of the Newcastle Medical Society, who, I suppose, heard his paper read, and afterwards kept it in his possession; for in 1796, Mr. T. Leighton writes to me thus. "In looking into authors on the subject of burns, I had found various stimulating applications recommended; but never having seen them used, I had not sufficient confidence to try any of them, until you recommended the *fluor volatile alkali* to me, and explained, in a very clear and candid manner, *your* ideas on the subject, and at the same time informed me of the result of your practice: I have ever since that period made use of the fluor volatile alkali, or oleum terebinth. sometimes one, and sometimes the other, and can hardly give a preference to either, both having the same good effects, (vide p. 175, first Essay.) From the above I think I am warranted in my conclusion, that Dr. Wood's paragraph, "We can apply, not only with safety, but with the best effect, ardent spirits to a part burned or scalded," would have produced no more effect upon the general treatment of burns, than the sentences I have quoted of Heister and of Sydenham. The fact of the cow-pock being a preventive against the small-pox, had been observed in different counties of England; but that did not lessen Dr. Jenner's merit in making public the fact, by which the attention of the rulers of nations was directed to it, and the inhabitants of the most distant parts of the world are likely to be affected by it. The above will shew, that although "some of the first surgeons at Newcastle" took the "*hint*" from Dr. Wood's publication, it had proved no hint to others; and again I disclaim all previous knowledge of Dr. Wood's work, except as a matter of conversation.

E. KENTISH, M. D.

*Bristol, June, 14th* 1808.

